# Extra Virgin Olive Oil Polyphenol-Enriched Extracts Exert Antioxidant and Anti-Inflammatory Effects on Peripheral Blood Mononuclear Cells from Rheumatoid Arthritis Patients

**DOI:** 10.3390/antiox14020171

**Published:** 2025-01-31

**Authors:** Bartolo Tamburini, Diana Di Liberto, Giovanni Pratelli, Chiara Rizzo, Lidia La Barbera, Marianna Lauricella, Daniela Carlisi, Antonella Maggio, Antonio Palumbo Piccionello, Antonella D’Anneo, Nadia Caccamo, Giuliana Guggino

**Affiliations:** 1 Department of Biomedicine, Neurosciences and Advanced Diagnostics (BIND), Section of Immunology, University of Palermo, 90127 Palermo, Italy; bartolo.tamburini@unipa.it (B.T.); nadia.caccamo@unipa.it (N.C.); 2 Department of Health Promotion, Mother and Child Care, Internal Medicine and Medical Specialties, Rheumatology Section, University Hospital “P. Giaccone”, 90127 Palermo, Italy; chiara.rizzo06@unipa.it (C.R.); lidia.labarbera@unipa.it (L.L.B.); giuliana.guggino@unipa.it (G.G.); 3 Department of Biomedicine, Neurosciences and Advanced Diagnostics (BIND), Section of Biochemistry, University of Palermo, 90127 Palermo, Italy; diana.diliberto@unipa.it (D.D.L.); giovanni.pratelli@unipa.it (G.P.); daniela.carlisi@unipa.it (D.C.); 4 Department of Biological, Chemical and Pharmaceutical Sciences and Technologies (STEBICEF), University of Palermo, Viale delle Scienze, 90128 Palermo, Italy; antonella.maggio@unipa.it (A.M.); antonio.palumbopiccionello@unipa.it (A.P.P.); 5 Department of Biological, Chemical and Pharmaceutical Sciences and Technologies (STEBICEF), Laboratory of Biochemistry, University of Palermo, 90127 Palermo, Italy; antonella.danneo@unipa.it

**Keywords:** rheumatoid arthritis, PBMCs, extra virgin olive oil polyphenols, IL-1β, TNF-α, Nrf2

## Abstract

Rheumatoid arthritis (RA) is a long-term systemic autoimmune disorder that causes joint inflammation, swelling, pain, bone erosion, and deformities. Recent findings emphasize the anti-inflammatory and antioxidant properties of bioactive natural compounds, such as polyphenols extracted from plants and fruits, and their possible synergistic effect when used in combination with current therapies to improve the prognosis and symptoms of inflammatory rheumatic diseases. Here, we report that Sicilian extra virgin olive oil polyphenol-enriched extracts (PE-EVOOs) reduce intracellular reactive oxygen species (ROS) and pro-inflammatory cytokines, such as tumor necrosis factor-α (TNF-α) and interleukin-1 β (IL-1β), in peripheral mononuclear cells (PBMCs) obtained from both RA patients and healthy subjects (HSs) treated with lipopolysaccharides (LPS) as a control. HPLC-ESI-MS analysis highlighted that PE-EVOOs are rich in different polyphenolic compounds responsible for many of the observed biological effects. At molecular levels, Western blotting analyses revealed that PE-EVOO treatment is associated with the downregulation of the phosphorylated and active form of the inflammatory transcription factor NF-κB and the pro-inflammatory enzyme cyclooxygenase 2 (COX2). In addition, PE-EVOOs upregulated the transcription factor Nrf2 and its target antioxidant enzyme catalase and manganese superoxide dismutase (MnSOD). Collectively, these results suggest a possible use of PE-EVOOs as potential adjuvants for the treatment of RA.

## 1. Introduction

Rheumatoid arthritis (RA) is a chronic systemic autoimmune disease primarily causing synovial joint inflammation, cartilage devastation, bone erosion, pain, deformities, and progressive disability with a reduction in an individual’s quality of life [1]. Although its etiopathogenesis is still unclear, it is estimated that RA affects 0.5–1% of the global population [2]. Current management strategy primarily focuses on the reduction in symptoms at the joints and the slowdown of its progression towards disability through the administration of disease-modifying anti-rheumatic drugs (DMARDs) either alone or in association with nonsteroidal anti-inflammatory drugs (NSAIDs) or glucocorticoids [3]. DMARDs are immunosuppressive and immunomodulatory agents classified as conventional DMARDs, such as methotrexate, leflunomide, hydroxychloroquine, and sulfasalazine, biologic DMARDs, introduced in the early nineties and, more recently, the synthetic target molecules usually prescribed after the failure of conventional DMARD therapy. They are monoclonal, chimeric humanized fusion antibodies, receptors fused to a part of the human immunoglobulin, or small molecules, like Janus kinase (JAK) inhibitors, highly specific and targeting a specific pathway of the immune system [4]. Although the availability of biological DMARDs has significantly improved the course of the disease in patients suffering from RA, patients often do not adequately respond to current treatment regimens due to tolerance development or severe side effects. Thus, over the past few decades, researchers have investigated if natural phytochemicals are effective in relieving RA-associated symptoms. Several phytochemicals, such as alkaloids, flavonoids, steroids, terpenoids, and polyphenols have shown anti-inflammatory and immunomodulatory activity against RA [5,6,7]. However, their use is often limited because of their high molecular weight, poor water solubility, permeability, and stability affecting their absorption and bioavailability, which can be currently implemented by phenolic-enabled nanotechnology (PEN) [8,9]. The Mediterranean diet is a healthy eating plan characterized by the high consumption of fruits, vegetables, and whole grains and reduced consumption of red meat. Extra virgin olive oil (EVOO), the main source of Mediterranean diet lipids, is obtained via mechanical extraction from the olive fruit under conditions that do not alter its composition. The consumption of EVOO has been shown to exert beneficial effects against several immune-inflammatory chronic diseases, including RA, systemic lupus erythematosus (SLE), and inflammatory bowel disease (IBD) [10,11,12].

In the past, the health-promoting properties of EVOO have been largely correlated to the high content of monounsaturated fatty acids, in particular oleic acid. However, more recently, attention has been focused on the presence of a variety of phenolic compounds, such as hydroxytyrosol (HTy), tyrosol, and oleuropein [13], on which the pharmacological properties of the olive tree seem to depend [14,15]. Thus, EVOO quality depends not only on the content of free fatty acids but also its content in polyphenols. Polyphenols are a class of compounds characterized by the presence of one or more aromatic rings and two or more hydroxyl groups. They are found in different parts of many plants including roots, stems, leaves, fruit, and flowers and are produced for defense against pathogens (microbes and fungi) and to discourage leaf-eating insects [16]. Due to their structure, they possess antioxidant and anti-inflammatory properties and are able to modulate abnormal cellular signaling induced by pro-inflammatory stimuli and oxidative stress, such as those related to the nuclear factor kappa-light-chain enhancer of activated B cells (NF-κB) and NF-E2-related factor 2 (Nrf2) [17].

Several recent studies [18] demonstrated the ability of EVOO polyphenols to activate the signaling pathway of Nrf2, a transcription factor that regulates the expression of phase II detoxifying enzymes, including NAD(P)H quinone oxidoreductase 1, glutathione peroxidase, ferritin, heme oxygenase-1 (HO-1), and antioxidant genes, which protect cells from different injuries via their anti-inflammatory effects, thus influencing the course of several diseases characterized by inflammation, such as RA, asthma, IBD, and Helicobacter pylori infection-induced gastritis [19]. Furthermore, EVOO polyphenols exert their anti-inflammatory activity also by preventing the activation of NF-κB, a protein complex responsible for the transcription of pro-inflammatory mediators, such as interleukin-6 (IL-6), tumor necrosis factor-α (TNF-α), inducible nitric oxide synthase (iNOS), interleukin-1 β (IL-1β), and cyclooxygenase 2 (COX2), in almost all types of animal cells and are involved in different processes such as inflammation, oxidative stress, apoptosis, immune response, cell growth, and development [17].

The consumption of EVOO or EVOO polyphenol-enriched extracts (PE-EVOOs) exerts beneficial effects against RA [20]. In collagen-induced arthritis (CIA), DBA/1 mice treated with EVOO or PE-EVOO have been shown to reduce joint edema and cartilage degradation, as well as decrease the levels of pro-inflammatory cytokines, preventing arthritis development [20,21]. In addition, in IL-1β-activated human synovial SW982 fibroblasts PE-EVOOs inhibited the production of pro-inflammatory mediators, like matrix metalloproteinases 1 (MMP-1) and IL-6, and this protective effect seems to be related to the inhibition of mitogen-activated protein kinases (MAPKs) (JNK and p38), which are involved in NF-κB activation in the cytoplasm and the modulation of its transactivating potential in the nucleus [22,23,24]. Here, we investigated the modulation of the inflammatory response in peripheral blood mononuclear cells (PBMCs) isolated from RA by treatment with PE-EVOOs. Our results provided evidence that PE-EVOOs exhibit significant anti-inflammatory properties by inhibiting NF-κB activation and downregulating the levels of COX-2 and the pro-inflammatory cytokines TNF-α and IL-1β. Furthermore, PE-EVOOs displayed potent antioxidant effects, which appear to be mediated by the activation of Nrf2, the master regulator of cellular antioxidant defenses, and its targets manganese superoxide dismutase (MnSOD) and catalase.

## 2. Materials and Methods

### 2.1. Chemical Extraction of Polyphenols from EVOO

EVOO was purchased from Azienda Agricola Signorello, Campobello di Mazzara, Sicily, Italy. PE-EVOOs were obtained through repeated centrifugations in a mixture containing ethanol and water at an 8:2 ratio, along with an immiscible n-hexane at a 1:1 ratio with the initial quantity of EVOO. Subsequently, the extracted polyphenol content was assessed using the Folin–Chiocalteu protocol [25] by determining the equivalent amount of gallic acid, one of the most represented polyphenols in EVOO [25]. To remove the ethanol, which can be toxic to cells, a freeze-drying process was employed, and the product obtained was resuspended in dimethyl sulfoxide (DMSO).

### 2.2. HPLC/MS/ESI/Q-Tof Analysis

Previously reported methods were adapted for performing HPLC/MS analysis [26,27]. A solution of the freeze-dried sample in ethanol (1 mg/mL *v*/*v*) was prepared. The ethanolic solution was directly injected without further treatment. Water and acetonitrile were of HPLC/MS grade. Formic acid was of analytical quality. A reversed-phase Phenomenex Luna Omega Polar C18 100 column, (150 mm × 4.6 mm, particle size 5 µm) with a Phenomenex (Torrance, CA 90501-1430, USA) C18 security guard column (4 × 3 mm) was used. The injection volume was 25 µL. The eluate was monitored with Mass Total Ion Count (MS TIC) and UV (530 nm). Mass spectra were obtained on an Agilent 6540 UHD, Santa Clara, CA 95051, USA, accurate-mass quadrupole time-of-flight (Q-TOF) spectrometer equipped with a dual AJS Electrospray Ionization (ESI) source working in negative mode. Nitrogen N2 was used as desolvation gas at 300 °C and a flow rate of 8 L min^−1^. The nebulizer was set to 45 psig. The sheath gas temperature was set at 400 °C and a flow of 12 L min^−1^. A potential 2.6 kV was used on the capillary. The fragmentor was set to 75 V. MS spectra were recorded in the 100–1500 m/z range. Quality control was performed prior to analysis using mass calibration in the range of 100–3000 Dalton (Q-TOF calibration mix) and solvent delay calibration for retention time. An in-house quality check mix containing known compounds (phenylalanine, saccharose, benzoic acid, and rutin) was injected during the batch of analysis. Mass spectrum data were analyzed for metabolite annotation using MassHunter Qualitative Analysis B.06.00 and the Metabolomics Workbench database (https://www.metabolomicsworkbench.org/) (accessed on 1 December 2024).

### 2.3. Patients

For our study, 22 RA naïve patients were recruited at the Rheumatology Unit of Policlinico “Paolo Giaccone”, University of Palermo. All patients recruited met the ACR/EULAR 2010 classification criteria for RA. They had active diseases and had never received DMARD treatment. Patients had previously been treated with a stable dose of NSAIDs. All patients presented active diseases, defined as a disease activity score of 28 C-reactive protein (DAS28CRP) > 5.1. In addition, 15 healthy subjects (HSs) were recruited as controls, paired by age and sex. The baseline demographic and clinical features of the patients are shown in Table 1. This study was approved by the local Ethics Committee of the University of Palermo and complied with the provisions of the Declaration of Helsinki. Informed consent was obtained from all patients and control.

### 2.4. MTS Assay

The MTS assay (CellTiter 96^®^ AQueous One Solution Cell Proliferation Assay, MTS, Promega Corporation, Madison, WI, USA) was used to evaluate the metabolic activity and viability of PBMCs. The assay involves the conversion of the tetrazolium salt MTS (3-(4,5-dimethylthiazol-2-yl)-5-(3-carboxymethoxyphenyl)-2-(4-sulfophenyl)-2H-tetrazolium) to a purple formazan in the presence of phenazine methosulfate. The enzymes responsible are NADPH-dependent dehydrogenases, which are active only in viable cells. The absorbance of the resulting formazan solution is proportional to the number of viable cells and can be quantified using a spectrophotometer (Abs measured at 490–500 nm). To this end, PBMCs of HSs were incubated in a 96 multiwell plate (100 × 10^3^/well/100 μL) for 48 h with different doses (0.5–50 µg/mL) of PE-EVOOs. Then, 20 µL of MTS solution was added to each well at a final concentration of 0.33 mg/mL, incubated for 2 h at 37 °C, and the absorbance was recorded at 490 nm.

### 2.5. Radical Scavenging Activity by DPPH

Radical scavenging activity of PE-EVOOs was determined by DPPH (1,1-diphenyl-2-picrylhydrazyl) radical (Sigma, Merck KGaA, Darmstadt, Germany) [28]. Different amounts of PE-EVOOs (0.5–50 µg/mL) were added to 1 mL of ethanol DPPH solution (100 µM) and incubated for half an hour at room temperature and in the dark. DPPH radicals are stable molecules, characterized by a deep-violet color with an absorption maximum of 517, which is reduced by the addition of antioxidant compounds. Discoloration was measured spectrophotometrically. Ethanol DPPH solution was used to prepare the negative control (A0), and a blank sample (A2) containing ethanol was used as a reference. The radical scavenging activity (% of DPPH radical inhibition) was calculated using the following equation:

Inhibition (%) = 1 − (A1 − A2/A0 − A2) × 100

### 2.6. Isolation of PBMCs

Peripheral blood samples were obtained from healthy subjects (HSs) and RA patients and collected in ethylenediaminetetraacetic acid (EDTA) tubes. PBMCs were isolated from patients and HSs by density gradient centrifugation. Peripheral blood was diluted at a 1:2 ratio with PBS and then centrifuged on a Ficoll gradient (Lympholyte for human cell separation from Cederlane, Burlington, Ontario, Canada) in a 15 mL polystyrene conical centrifuge tube for 20 min at 770× *g* at room temperature (RT). Subsequently, PBMCs were carefully aspirated from the plasma–Ficoll interface and washed twice (at 500× *g* for 5 min) with fresh RPMI 1640 medium (Euroclone, Pero, Italy) supplemented with 20 mM HEPES, 100 U/mL penicillin, and 100 μg/mL streptomycin (Euroclone). Finally, PBMCs were resuspended in 1 mL of complete RPMI medium, containing 10% heat-inactivated FCS and 2 mM L-glutamine (Euroclone).

### 2.7. Cytofluorimetric Analysis

Following the Ficoll–Hypaque gradient separation, freshly isolated PBMCs from HSs and RA patients were cultured in a complete medium alone or in the presence of 10 μg/mL of PE-EVOO for 48 h. LPS (Sigma) (5 μg/mL) was added only to HS PBMCs cultured in complete medium alone or in the presence of PE-EVOOs (10 μg/mL) after the first 24 h of PE-EVOO treatment. In addition, 10 μg/mL of Monensin (BioLegend, San Diego, CA, USA) was added to all the conditions after the first hour of incubation as a Golgi blocker to ensure the intracellular retention of cytokines. At the end of the incubation period, PBMCs were stained with a Zombie NIR Fixable Viability Kit (BioLegend), a fluorescent dye to assess their viability, and anti-human monoclonal antibodies (mAbs) specific for surface molecules, such as CD3 PerCP clone REA613, CD4 PECy7 clone REA623, and CD14 APC clone REA599 (Miltenyi Biotec, Bergisch Gladbach, Germany), as previously reported [29,30]. Following surface molecule detection, PBMCs were fixed, permeabilized (Inside Stain Kit, Miltenyi Biotec), and incubated with mAbs directed against TNF-α FITC clone REA656 and IL-1β PE clone REA1172 (Miltenyi Biotec). Flow cytometry acquisition of stained PBMCs was carried out using the FACS Lyric cytometer (BD Biosciences, San Diego, CA, USA), and a minimum of 200,000 total events were acquired. Subsequently, sample analysis was performed using FlowJo v10 software (BD Biosciences).

### 2.8. Intracellular Detection of Reactive Oxygen Species (ROS) Through H_2_DCFDA Staining

Intracellular levels of reactive oxygen species (ROS) were assessed by quantifying the oxidation of the cell-permeable dye 2′,7′-dichlorodihydrofluorescein diacetate (H_2_DCFDA) (Molecular Probe, Life Technologies, Eugene, OR, USA) [28,31]. PBMCs obtained from RA patients and HSs were treated as previously described for cytofluorimetric analysis but in the absence of Golgi blocker Monensin. Subsequently, the cells, after washing in PBS, were incubated in the dark at 37 °C with 5% CO_2_ in the presence of 0.5 μM H_2_DCFDA dye for 5 min. Following the incubation, excess fluorochrome was removed through PBS washing, and the intracellularly produced fluorescent 2′,7′-dichlorofluorescein (DCF), resulting from oxidation, was quantified using flow cytometric assays. These assays employed excitation and emission wavelengths suitable for green fluorescence, with an FITC filter having an excitation wavelength of 488 nm and an emission wavelength of 530 nm.

### 2.9. Western Blotting Analysis

Western blot analyses were performed as previously reported [32,33,34]. Following treatment with LPS and/or PE-EVOOs, cells were rinsed with PBS and subsequently lysed at 4 °C for 30 min in an ice-cold lysis buffer containing 1% NP-40, 0.1% SDS, and 0.5% sodium deoxycholate in PBS and supplemented with a protease inhibitor cocktail. The lysate was then sonicated in three 10 s bursts. Protein concentration was determined using the Bradford protein assay (Bio-Rad Laboratories S.r.l., Segrate, Milan, Italy). Subsequently, an equivalent amount of proteins (30 μg/lane) was loaded and subjected to sodium dodecyl sulfate–polyacrylamide gel electrophoresis (SDS-PAGE). The gels were then transferred onto a nitrocellulose membrane (Bio-Rad). Immunodetection was carried out by incubating the membranes with specific primary antibodies: phosphorylated p65 NF-κB (Ser536, 93H1, Cell Signaling (Danvers, MA, USA)), p65 NF-κB (sc-8008, Santa Cruz, CA, USA), phosphorylated Nrf2 (pSer40, NBP2–67465, Santa Cruz, CA, USA), Nrf2 (NBP1-32822, Novus Biologicals Bio-Techne SRL, Milan, Italy), MnSOD (sc-133254, Santa Cruz, CA, USA), catalase (sc-271358, Santa Cruz, CA, USA), and COX2 (sc-7951, Santa Cruz, CA, USA). Finally, immunoreactive signals, developed using HRP-conjugated secondary antibodies (Amersham, GE Healthcare Life Science, Milan, Italy), were visualized with enhanced chemiluminescence (ECL) reagents (Cyanagen, Bologna, Italy) and captured with ChemiDoc XRS (Bio-Rad). Signal quantification was performed using Quantity One 1-D software. The intensity of the protein of interest was quantified via densitometric analysis using SMX Image software (Bio-Rad). Protein expression was normalized with GAPDH (AM4300, Invitrogen-TermoFisher Scientific, Milan, Italy) as a control.

### 2.10. Statistical Analysis

Multiple unpaired Student’s *t*-tests by GraphPad Prism, version 9.0 (GraphPad Software, San Diego, CA, USA), were used to perform statistical analyses reported here, and all the data were expressed as mean ± SD. A *p*-value of <0.05 was considered statistically significant for each test. When not specified, the data are not significant with respect to the related control. All experiments were conducted in triplicate.

## 3. Results

### 3.1. Characterization of Phytochemical Compounds Present in Sicilian EVOO Extracts

The phenolic composition of EVOO is profoundly influenced by agronomic, technological, and storage conditions. Factors such as olive cultivar, ripening stage, environmental conditions, harvesting methods, and milling techniques contribute to the diversity and concentration of phenolic compounds. In order to assess the composition of EVOO cultivated in the Mediterranean area (Sicily, Italy), ethanol extracts prepared as reported in the Section 2 were analyzed by an HPLC/ESI/Q-TOF in negative mode analysis. The analysis permitted the identification of 31 metabolites in ethanolic extracts of EVOO (Table 2). Approximately half of these metabolites belong to the saponifiable fraction, including carboxylic acids (mandelic acid and azelaic acid), fatty acids (oleic, linoleic, stearic, and palmitic acids), and fatty acid derivatives. The remaining metabolites were part of the unsaponifiable fraction, with derivatives of 3-hydroxytyrosol (3,4-dihydroxyphenylethanol, 3,4-DHPEA) emerging as the predominant compounds in the polar phenol extract. Ligstroside, formed by ester bonds between 3,4-DHPEA and oleanolic acid or its demethylated form, is one of two secoiridoids dominant in polar phenol extracts.

### 3.2. PE-EVOOs Possess Radical Scavenging Activity

**Figure 1:**
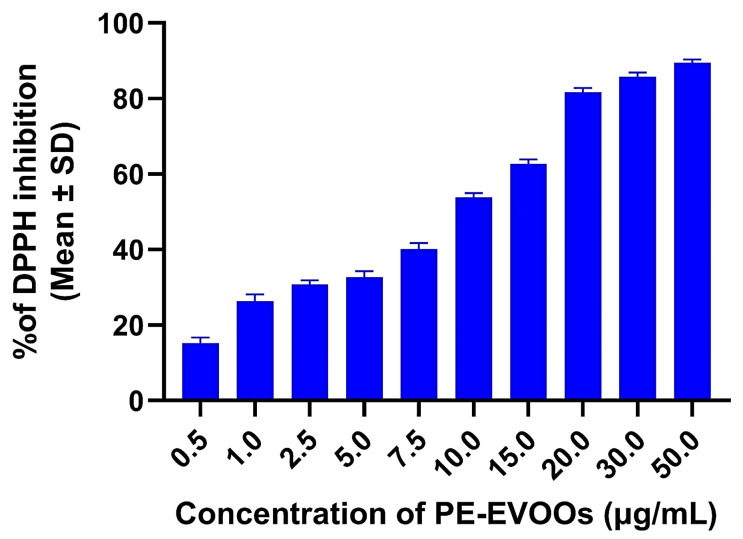
PE-EVOOs exert a high dose-dependent radical scavenging activity. The antioxidant activity of Sicilian PE-EVOOs was evaluated by a DPPH radical scavenging assay. Different concentrations of PE-EVOOs (from 0.5 to 50 µg/mL) were added to an ethanol DPPH• solution, and the relative absorption was measured at 517 nm spectrophotometrically. The bar graphs represent the mean of three independent experiments ± SD. *p*-value summary < 0.001 compared to the only vehicle control (ethanol).

To ascertain the potential radical scavenging activity of PE-EVOOs, different concentrations of the extracts (0.5–50 µg/mL) were incubated with DPPH, as reported in the Section 2. As shown in Figure 1, our data demonstrated that PE-EVOOs own a high and dose-dependent radical scavenging activity. Indeed, at the lower tested concentration (0.5 μg/mL), PE-EVOOs inhibited DPPH radicals by only 15%. This effect rises by increasing the dose of PE-EVOOs, reaching an inhibition value of 90% at 50 μg/mL.

### 3.3. Effects of PE-EVOOs on PBMC Viability

**Figure 2:**
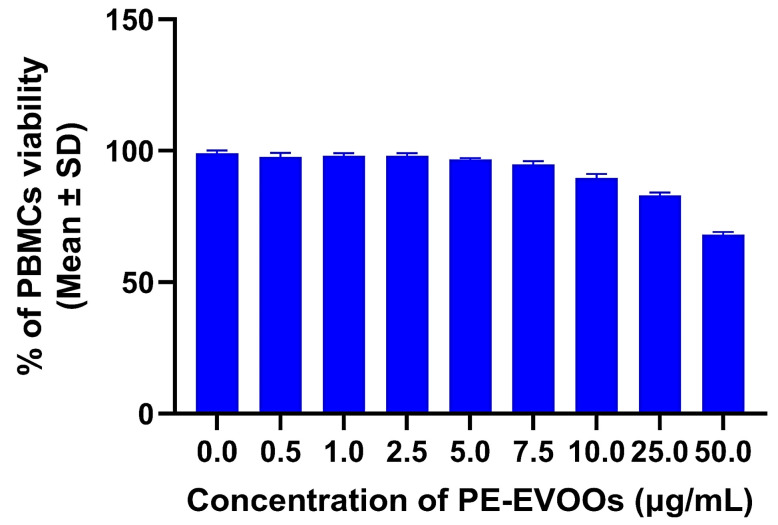
PE-EVOO effects on PBMC viability. PBMCs (100 × 10^3^/100 µL/well) were incubated with different doses (0.5–50 µg/mL) of PE-EVOOs for 48 h. Then, the percentage of viable cells was assessed by an MTS assay. The values reported are the mean ± SD of three independent experiments. *p*-value summary < 0.05 with respect to cells cultured in medium alone (RPMI).

The present study aimed to investigate whether PE-EVOOs were capable of counteracting oxidative stress and inflammatory pathways in RA patients’ PBMCs. To rule out potential cytotoxic effects of PE-EVOOs on PBMCs, preliminary experiments were performed by incubating HS PBMCs with different doses (0.5–50 µg/mL) of PE-EVOOs for 48 h. Then, the viability was evaluated by an MTS assay, as reported in the Section 2. The results reported in Figure 2 indicate that PE-EVOOs at 50 µg/mL reduced cell viability by 67%. However, doses of PE-EVOOs lower than 25 µg/mL did not affect PBMC viability.

Based on these preliminary data, the concentration of PE-EVOOs chosen for further experiments was 10 µg/mL, exhibiting 55% radical scavenging activity and a very low cytotoxic effect on PBMCs (about 10%).

### 3.4. PE-EVOOs Exert Anti-Inflammatory Effects in RA Patients and HS LPS-Stimulated PBMCs

**Figure 3:**
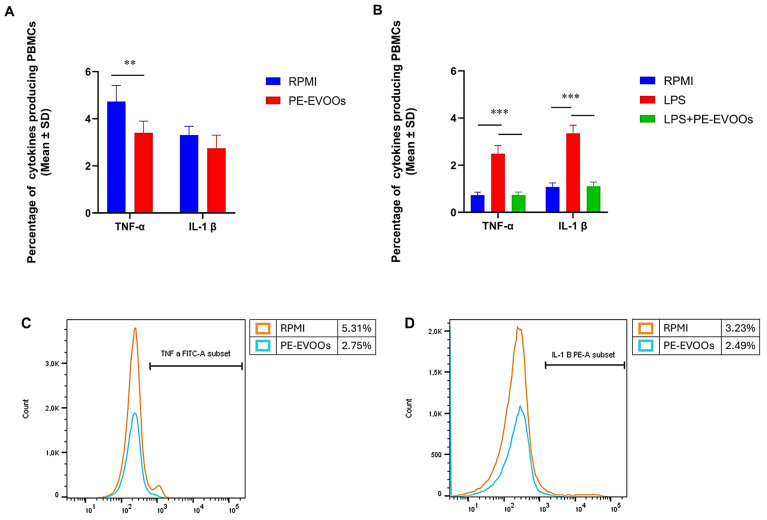
PE-EVOOs reduce the production of pro-inflammatory cytokines from RA patients and HS LPS-stimulated PBMCs. Cumulative data obtained from flow cytometric analyses of RA patients (**A**) and HS LPS-stimulated (**B**) PBMCs intracellularly stained for TNF-α and IL-1β show that both cytokines decreased after 48 h of 10 µg/mL PE-EVOO treatment. Histogram overlays of the production of TNF-α (**C**) and IL-1β (**D**) from an RA representative patient. ** *p* < 0.01 and *** *p* < 0.001 with respect to cells cultured in medium alone (RPMI) or with LPS (LPS).

Then, to investigate whether PE-EVOOs exert anti-inflammatory effects on RA patients’ PBMCs, we isolated them from RA patients, as described in the Section 2, and treated them in vitro for 48 h with 10 μg/mL of PE-EVOOs. After treatments, cytofluorimetric analyses were performed to evaluate the production by PBMCs of TNF-α and IL-1β, the main cytokines playing an important role in the development and progression of inflammatory rheumatic disease (IRDs). As shown by cumulative data of cytofluorimetric analyses obtained from RA patients and reported in Figure 3A, PE-EVOO treatment reduced the percentage of RA PBMCs producing both TNF-α and IL-1β with respect to untreated RA PBMCs. In fact, the mean percentage of TNF-α^+^ RA PBMCs lowered from 4.73 (SD ± 0.681) in untreated cells to 3.40 (SD ± 0.490) in PE-EVOO-treated ones. In addition, the percentage of IL-1β^+^ RA PBMCs decreased from 3.31 (SD ± 0.375) in untreated cells to 2.75 (SD ± 0.558) in PBMCs treated for 48 h with PE-EVOOs.

We also evaluated the effects of PE-EVOOs treated on HS PBMCs stimulated with 5 µg/mL of LPS, as reported in the Section 2, to simulate in vitro the inflammatory conditions that characterize RA patients. Interestingly, the anti-inflammatory effect of PE-EVOOs was also observed in LPS-treated HS PBMCs. In fact, in HS PBMCs, LPS treatment increased the production of both TNF-α and IL-1β, and these cytokines were both reduced in a statistically significant manner when the cells were pretreated with PE-EVOOs. In fact, as the same Figure 3B shows, the mean percentage for TNF-α^+^ cells was reduced from 2.49 (SD ± 0.347) in PBMCs stimulated with LPS alone to 0.73 (SD ± 0.129) in PE-EVOO-pretreated cells and for IL-1β^+^ cells from 3.35 (SD ± 0.334) in PBMCs stimulated with LPS alone to 1.11 (SD ± 0.174) in PE-EVOO-pretreated cells. Figure 3C,D show histogram overlays of the production of TNF-α and IL-1β, respectively, obtained from an RA-representative patient.

**Figure 4:**
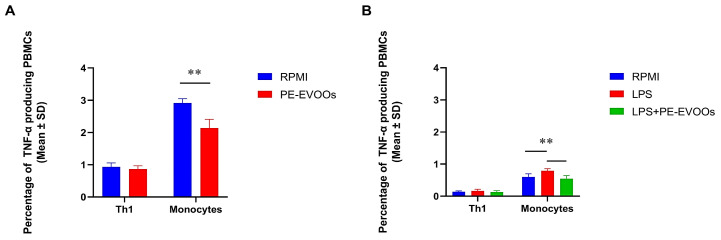
PE-EVOOs reduce TNF-α production only from monocytes and not from T helper cells. Cumulative data obtained from flow cytometric analyses of RA patients (**A**) and HS LPS-stimulated (**B**) PBMCs intracellularly stained for TNF-α production from Th1 cells and monocytes show that only monocytes producing TNF-α decreased after 48 h of 10 µg/mL PE-EVOO treatment. ** *p* < 0.01 with respect to cells cultured in medium alone (RPMI) or with LPS.

In addition, to understand which cell subset was more susceptible to the anti-inflammatory PE-EVOO effect, cytofluorimetric analyses were performed on RA patients and HSs to evaluate the effect of PE-EVOO treatment on TNF-α production from T helper cells and monocytes, characterized by different cell surface markers (CD3^+^/CD4^+^ for T helper subset and CD3^−^/CD14^+^ for monocytes). Our data reported in Figure 4A demonstrated that 48 h of treatment with 10 μg/mL of PE-EVOOs reduced the percentage of monocytes producing TNF-α in RA patients, with a mean percentage going from 2.91 (SD ± 0.130) in untreated PBMCs to 2.18 (SD ± 0.356) in PE-EVOO-treated PBMCs. The same effect was observed in HSs, where the percentage of monocytes producing TNF-α lowered from 0.79 (SD ± 0.065) in LPS-stimulated PBMCs to 0.54 (SD ± 0.097) in PE-EVOO-pretreated LPS-stimulated PBMCs (Figure 4B). Instead, the production of TNF-α from T helper cells remained unvaried in both RA and LPS-stimulated HS PBMCs.

### 3.5. PE-EVOO Treatment Reduces the Activation of NF-κB in RA Patients and HS LPS-Stimulated PBMCs

NF-κB is identified as one of the main transcription factors involved in the transcription of inflammatory cytokines, like IL-1β, TNF-α, and COX2, whose increased levels in RA are associated with bone erosion, pain hypersensitivity, and disease progression [35].

**Figure 5:**
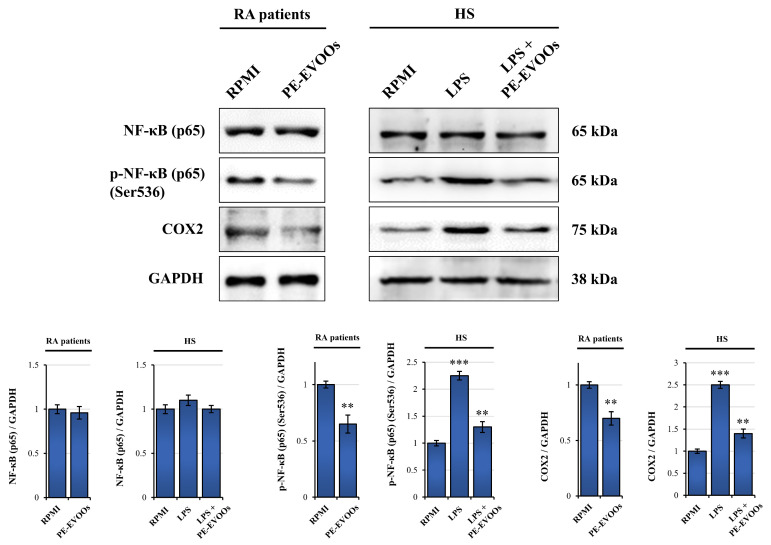
PE-EVOOs exert anti-inflammatory effects by reducing the expression of the active form of NF-κB and COX2. Western blotting analysis of NF-κB, phosphorylated-NF-κB, and COX2 in RA patients and HS LPS-stimulated PBMCs treated for 48 h with 10 µg/mL of PE-EVOOs. Equal loading of proteins was verified by immunoblotting for GAPDH. The bar graphs represent the mean of three independent experiments. ** *p* < 0.01 and *** *p* < 0.001 with respect to cells cultured in medium alone (RPMI).

In mammals, the NF-κB family consists of five members: RelA (p65), RelB, c-Rel, NF-kB1 (p50 and its precursor p105), and NF-κB2 (p52 and its precursor p100), which form different homodimers and heterodimers, and each of them activates its own characteristic target genes [36,37,38]. It has been reported that TNF-α induces via IKK the phosphorylation of p65 RelA on Ser-536, leading to the activation of NF-κB signaling and enhanced inflammation [39]. For this reason, we investigated the ability of PE-EVOOs to modulate the activity of phospo-NF-κB p65 (Ser536) in RA patients and HS LPS-stimulated PBMCs. The expression levels of both NF-κB and its phosphorylated p65 subunit were evaluated by Western blotting analysis in both RA patients and HS LPS-stimulated PBMCs alone or after 48 h of treatment with 10 µg/mL of PE-EVOOs. Our data demonstrated that the PE-EVOO treatment reduced the levels of phosphorylated NF-κB p65 in both RA patients and HS LPS-stimulated PBMCs, whereas the expression levels of total NF-κB did not change in both cases (Figure 5). Thus, the reduced activity of NF-κB could, in part, mediate the anti-inflammatory effect of PE-EVOOs.

Moreover, in RA patients and HS LPS-stimulated PBMCs, PE-EVOO treatment also reduced the expression levels of COX2, a pro-inflammatory enzyme whose expression is upregulated by the increased IL-1β secretion via NF-kB [40] (Figure 5).

### 3.6. PE-EVOOs Exert Antioxidative Effects in RA Patients and HS LPS-Stimulated PBMCs

It has been reported that ROS generation plays a key role in the pathogenesis of several IRDs, including RA [41]. It is now well established that elevated ROS production amplifies the inflammatory response by upregulating genes associated with immune and inflammatory cytokines through the activation of NF-κB, a nuclear transcription factor sensitive to redox signaling [42].

**Figure 6:**
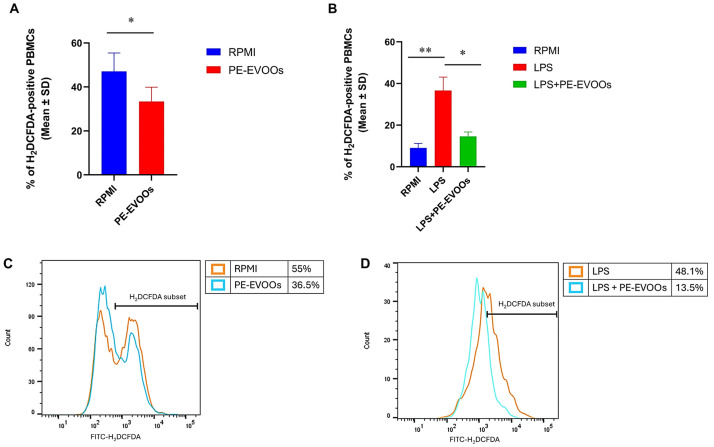
PE-EVOOs exert antioxidant effects in RA patients and HS LPS-stimulated PBMCs. Cumulative data obtained from flow cytometric analysis of RA patients (**A**) and HS LPS-stimulated (**B**) PBMCs treated for 48 h with 10 µg/mL of PE-EVOOs stained for intracellular ROS using the redox-sensitive fluorochrome H2-DCFDA, as reported in the Section 2. Histogram overlays of intracellular ROS production obtained from PBMCs of a representative RA patient (**C**) and HSs (**D**). * *p* < 0.05 and ** *p* < 0.01 with respect to cells cultured in medium alone (RPMI) or with LPS.

Therefore, we investigated the ability of PE-EVOOs to reduce ROS production in both RA and LPS-stimulated HS PBMCs. Cumulative cytofluorimetric analyses, obtained by the oxidation of the H2DCFDA dye, showed that 48 h of treatment with 10 µg/mL of PE-EVOOs caused a reduction in intracellular ROS in RA patients’ PBMCs, from a mean percentage of 47.14 (SD ± 8.322) H_2_DCFDA^+^ untreated PBMCs to 33.40 (SD ± 6.542) H2DCFDA^+^ PE-EVOOs treated PBMCs (Figure 6A). A similar effect was observed in HS PBMCs, where PE-EVOO treatment significantly reduced ROS production induced by LPS treatment (5 µg/mL) (Figure 6B). The mean percentage of H_2_DCFDA^+^ cells decreased from 36.68 (SD ± 14.268) in LPS-stimulated PBMCs to 14.60 (SD ± 4.771) in PE-EVOO-pretreated LPS-stimulated PBMCs (Figure 6B). Figure 6C,D show histogram overlays of intracellular ROS production obtained from PBMCs of a representative RA patient and HSs, respectively.

### 3.7. PE-EVOO Treatment Upregulates the Antioxidant Nrf2-Mediated Pathway in RA Patients and HS LPS-Stimulated PBMCs

**Figure 7:**
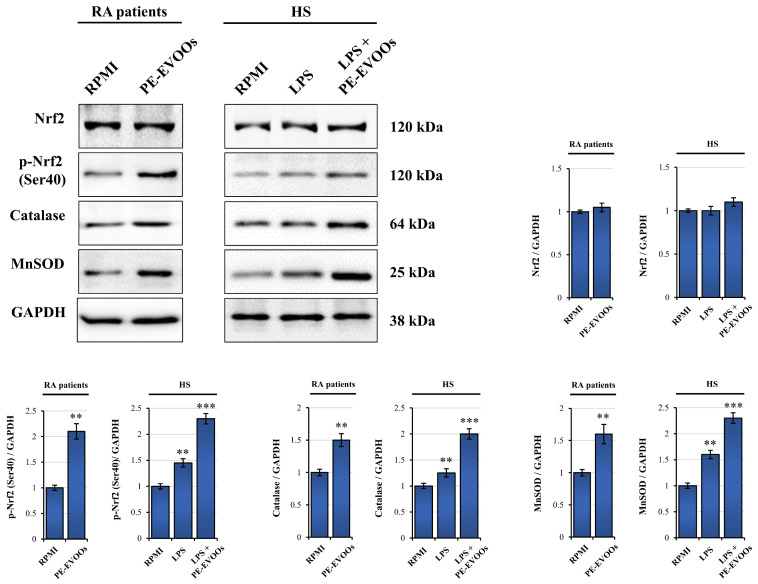
PE-EVOOs exert antioxidant effects upregulating the active form of Nrf2 and its transcriptional targets, catalase and MnSOD. Western blotting analysis of Nrf2, p-Nrf2 (Ser 40), catalase, and MnSOD in RA patients and HS LPS-stimulated PBMCs treated for 48 h with 10 µg/mL of PE-EVOOs. Equal loading of proteins was verified by immunoblotting for GAPDH. The bar graphs represent the mean of three independent experiments ± SD. ** *p* < 0.01 and *** *p* < 0.001 with respect to cells cultured in medium alone (RPMI).

To deeply investigate the molecular mechanisms responsible for PE-EVOO antioxidant effect on RA patients and HSs, we performed Western blotting experiments to evaluate the ability of PE-EVOOs to upregulate Nrf2, a transcription factor that controls the expression of several antioxidant genes, regulating the physiological and pathophysiological outcomes of oxidant exposure [43]. We demonstrated that PE-EVOO treatment increased the levels of phosphorylated and active forms of Nrf2 in RA patients and HS LPS-stimulated PBMCs, while the levels of total Nrf2 did not change (Figure 7).

According to these data, we further demonstrated by Western blotting analysis that the expression levels of catalase and MnSOD, two important ROS scavenger enzymes regulated transcriptionally by Nrf2 [44], markedly increased in PBMCs from RA patients and HSs stimulated with LPS after 48 h of PE-EVOO treatment.

Taken together, these results seem to indicate that the activation of Nrf2 and antioxidant enzymes, catalase and MnSOD, could mediate the antioxidant behavior of PE-EVOOs in both RA patients and HS LPS-stimulated PBMCs.

## 4. Discussion

RA is one of the most prevalent complex, chronic, inflammatory diseases, manifested by elevated oxidative stress and inflammatory biomarkers [45]. While current RA therapies, including conventional DMARDs, biologics, and corticosteroids, have significantly improved the course of the disease, there is an urgent need for novel approaches that can better target the underlying pathogenetic mechanisms, reduce side effects, prevent joint damage, and improve long-term quality of life [46]. Recently, several natural compounds have been shown to exert a supportive role in managing RA, helping to reduce inflammation, relieve pain, and potentially improve joint health [21].

As part of the ongoing quest to identify natural compounds for the treatment of RA, our study focused on investigating the anti-inflammatory and antioxidant properties of polyphenol-rich extracts derived from extra virgin olive oil produced in Sicily, Italy. This region, located in the Mediterranean basin, is characterized by an optimal climate, traditional olive harvesting techniques, and the immediate milling of olives after harvest, all of which contribute to producing high-quality EVOO with a superior nutritional and chemical profile. Notably, the DPPH assay evidenced that ethanol extracts of Sicilian EVOO possess strong ROS scavenging properties. The characterization of the polyphenolic profile of these extracts using HPLC/MS provided evidence that they are rich in polyphenols. In particular, we demonstrated the presence of 3,4-DHPEA and its derivatives. The high prevalence of 3,4-DHPEA and its derivatives underscores the significant role these compounds play in EVOO’s biological effects. The potent antioxidant activity of 3,4-DHPEA is well documented, with mechanisms such as free radical scavenging and metal ion chelation supporting its cardioprotective, neuroprotective, and anticancer effects [47,48]. While the antioxidant activity forms the foundation of its health benefits, the molecular pathways underlying its diverse biological effects warrant further exploration [15].

TNF-α, IL-1β, and IL-6 are pro-inflammatory cytokines that play a critical role in the pathogenesis and progression of RA, with growth-promoting properties directed towards synovial fibroblasts whose hyperproliferation is responsible for the alteration of joint structures and destruction of connective tissue and subchondral bone [49]. Therefore, elevated serum levels of these cytokines are commonly observed in individuals with active RA and are associated with disease severity and joint damage [50]. In line with this observation, we demonstrated that RA PBMCs expressed elevated levels of both TNF-α and IL-1β. Notably, an increased expression of both pro-inflammatory cytokines was observed in healthy-subject PBMCs stimulated with lipopolysaccharide LPS to mimic an inflammatory condition. Notably, RA PBMCs were not stimulated with LPS because it is widely reported that the high basal secretion of pro-inflammatory cytokines characterizing RA patients causes a decrease in the ability to secrete increased amounts of cytokines in response to pro-inflammatory stimulation, such as LPS [51].

High levels of circulating TNF-α, produced primarily by activated macrophages and T cells and, to a lesser extent, by neutrophils, mast cells, and endothelial cells [52], lead to fibroblast activation in RA patients, resulting in the recruitment of inflammatory cells into the lesion and the NF-κB-mediated secretion of inflammatory cytokines, cathepsins, matrix metalloproteinases, and other inflammatory mediators, which cause destructive changes in the joints [53]. TNF-α binds to the ubiquitously expressed TNF receptor 1 (TNFR1/TNFR-55) or the tissue-restricted TNFR2 (TNFR-75) [54]. TNFR1 binding activates canonical NF-κB, the MAP kinases p38 and JNK, and due to the presence of a death domain, apical caspases. Signaling via TNFR2, which does not contain a death domain, activates MAP kinases and also leads to noncanonical NF-κB activation [54,55], causing the differentiation of multiple Th subsets towards inflammatory phenotypes, like Th1 and Th17.

IL-1β is a member of the interleukin-1 family, produced by activated macrophages, monocytes, and a subpopulation of dendritic cells called slanDC, as a proprotein, which is proteolytically processed into its active form by caspase 1 (CASP1/ICE) [56]. IL-1β is a central mediator of innate immunity and inflammation, leading to fever onset and immune activation upon binding to IL-1 receptor 1, and its secretion is tightly regulated.

The activation of Toll-like receptors (TLRs), TNF-α receptors, or IL-1 receptors upon binding of mature IL-1α or IL-1β, induces the transcription of the biologically inactive pro-form, although for its secretion, inflammasome activation is requested [56,57]. IL-1β, together with TNF-α and IL-6 serum levels, often correlate with disease severity, including joint swelling and bone erosion [58,59]. For this reason, a reduction in the synthesis of both TNF-α and IL-1 β using bioactive natural compounds may represent an effective method to manage inflammation in IRDs, including RA [60].

Interestingly, we found that exposure to PE-EVOOs significantly reduced the production of TNF-α and IL-1β, both in RA PBMCs and in HS PBMCs stimulated with LPS, thus suggesting an anti-inflammatory potential of EVOO extracts. In addition, the observation that the treatment with PE-EVOOs efficiently reduces specifically monocytic TNF-α production confirms that their use could be beneficial to RA symptoms, given its role in cartilage and bone infiltration and destruction characterizing RA progression. Our findings in fact support the literature data reporting that TNF-α-producing macrophages are the most abundant immune cells found in RA synovium, where they produce also other pro-inflammatory cytokines (IL-1β and IL-6), chemoattractant factors (CCL2 and IL-8), and metalloproteinases (MMP-3 and MMP-12) [61,62,63], inducing cellular proliferation and increased vascular permeability, which contribute to the pathogenic features of tissue proliferation and neovascularization characterizing rheumatoid synovium, along with cartilage and bone infiltration and destruction [63].

It has been known that IL-1β upregulates the expression of COX2, an inducible enzyme that converts arachidonic acid into prostaglandins, amplifying the inflammatory response [64]. In turn, COX2-derived prostaglandins, like PGE2, can enhance the release of IL-1β from immune cells, creating a feedback loop that sustains inflammation [65]. In line with these suggestions, the reduction in COX2 production observed in RA PBMC treated with PE-EVOOs may contribute to reduced inflammation in RA patients.

Our data also demonstrated that PE-EVOOs reduced the active phosphorylated form of NF-κB in both RA and healthy LPS-stimulated PBMCs. NF-κB is a critical transcription factor that controls the expression of a wide array of pro-inflammatory genes [65]. Its activation is promoted by different pro-inflammatory stimuli, like TNF-α or IL-1β, or TLR activation by pathogens [66]. When activated, NF-κB binds to specific DNA response elements in the nucleus, initiating the transcription of pro-inflammatory genes, including TNF-α and other cytokines, such as IL-1β and IL-6, in a positive feedback loop [67]. Thus, the reduction in NF-κB activation may explain the anti-inflammatory role exerted by PE-EVOOs in RA PBMCs.

A link between chronic inflammation and oxidative stress has been observed, with free radicals being the cause of many chronic diseases, such as RA [68]. Sustained ROS production can lead to chronic NF-κB activation, contributing to inflammation and tissue damage [68]. ROS act as signaling molecules activating NF-κB by activating the IκB kinase (IKK) complex, which phosphorylates IκB (inhibitor of NF-κB), marking it for ubiquitination and proteasome 26S degradation [68]. This releases NF-κB, allowing it to translocate to the nucleus and activate target genes [68]. ROS can also directly oxidize specific cysteine residues on IKK or other upstream signaling proteins, modulating their activity [69]. Thus, the reduction in ROS level can contribute to inactivating NF-κB and inflammation. Notably, our data provided evidence that PE-EVOOs reduce the production of ROS both in RA PBMCs and HS LPS-stimulated PBMCs. The antioxidant effects of PE-EVOOs could be explained by the ability of these extracts to upregulate the transcriptional factor Nrf2 and its antioxidant target enzymes MnSOD and catalase in RA PBMCs and healthy LPS-stimulated PBMCs.

In line with our hypothesis, it has been shown that Nrf2 prevents LPS-induced transcriptional upregulation of several pro-inflammatory cytokines, such as IL-6 and IL-1β [70], whose production is increased in Nrf2−/− mice with dextran sulfate-induced colitis [70]. Nrf2 also inhibits the production of IL-17 and other Th1 cytokines, reducing disease progression in autoimmune encephalitis, an experimental model of multiple sclerosis [71]. Furthermore, Nrf2-dependent antioxidant genes, such as HO-1, NQO-1, Gclc, and Gclm, block the production of TNF-α, IL-6, monocyte chemo-attractant protein-1 (MCP1), macrophage inflammatory protein-2 (MIP2), and other inflammatory mediators [70,72]. Our results, therefore, underline how the marked anti-inflammatory activity of PE-EVOOs is strongly correlated to the reduction in oxidative stress through the increased expression of the active form of Nrf2 and its target antioxidant genes, MnSOD and catalase.

## 5. Conclusions

Our findings confirm the dual anti-inflammatory and antioxidant efficacy of PE-EVOOs, underscoring their potential as natural therapeutic agents for managing RA. The anti-inflammatory activity of PE-EVOOs seems to be mediated by the inhibition of the NF-κB signaling pathway. This suppression significantly reduces the expression of COX2, a pivotal enzyme in the inflammatory process, and decreases the synthesis of critical pro-inflammatory cytokines, such as TNF-α and IL-1β. This mechanism may help to mitigate the chronic inflammation associated with RA and other degenerative diseases. Simultaneously, PE-EVOOs exhibit robust antioxidant properties by neutralizing ROS and upregulating the expression of essential antioxidant enzymes, including catalase and MnSOD. This effect seems to involve the activation of Nrf2. By protecting cells from oxidative damage, which often exacerbates chronic inflammation, PE-EVOOs provide a comprehensive therapeutic benefit.

These results highlight the potential of PE-EVOOs not only to modulate inflammatory responses but also to enhance antioxidant defense mechanisms, suggesting a valuable role in the management of chronic inflammatory conditions, such as RA. By leveraging their natural bioactive properties, PE-EVOOs could serve as an adjunct to conventional RA therapies, contributing to pain relief, inflammation reduction, and improved overall treatment outcomes. This dual action underscores their promise as a complementary natural intervention for enhancing the quality of life in individuals with RA. Comprehensive clinical trials are needed to confirm the efficacy and safety of PE-EVOOs in diverse patient populations and to explore optimal dosing regimens. Such research will be crucial to translate these preliminary findings into viable therapeutic strategies, paving the way for their integration into evidence-based management plans for RA and other chronic inflammatory diseases.

## Figures and Tables

**Table 1:** Clinical characteristics of RA patients and HSs.

	RA (*n* = 22)	HSs (*n* = 15)
Age mean, years (range)	54.8 (32–70)	47.2 (31–60)
Female sex, *n* (%)	14 (63.6)	11 (73.3)
Disease duration, years (range)	3 (0.5–5.7)	-
Anti-CCP, *n* (%)	10 (45.5)	-
RF, *n* (%)	13 (59)	-
CRP mg/l, mean (range)	6.2 (0.2–7.6)	-
ESR, mean (range)	27.7 (1–89)	-
DAS28CRP score, mean (range)	3.4 (0.9–5.7)	-

Anti-CCP: cyclic citrullinated peptide; CRP: C-reactive protein; DAS28CRP: disease activity score 28CRP; ESR: erythrocyte sedimentation rate; HSs: healthy subjects; RA: rheumatoid arthritis; RF: rheumatoid factor.

**Table 2:** The bioactive natural compounds in the phenolic extracts of Sicilian EVOO. Metabolites were identified using HPLC-ESI-MS analysis.

	Compound	Rt (min)	ESI^+^ [M-H]^−^ (m/z) (*Exp*.)	ESI^−^ [M-H]^−^ (m/z) (*Teor*.)	Molecular Formula	Metabolite Classes
1	Mandelic acid	3.17	151.0417	151.0401	C_8_H_8_O_3_	Carboxylic acid
2	Abscisic acid	7.59	309.1368 ^a^	309.1344	C_15_H_20_O_4_	Phytohormone
3	Azelaic acid	9.19	187.1010	187.0976	C_9_H_16_O_4_	Carboxylic acid
4	3-hydroxytyrosol 4-glucoside	9.95	351.0801	351.0852	C_14_H_20_O_8_	Phenolic compound
5	Abscisic acid isomer	10.45	309.1368 ^a^	309.1344	C_15_H_20_O_4_	Phytohormone
6	Erythritol phosphate	13.82	201.0218	201.0170	C_4_H_11_O_7_P	Sugar derivative
7	Oleuropein aglycon	15.98	377.1276	377.1242	C_19_H_22_O_8_	Secoiridoid
8	5,6′-dihydroxy-6,7-dimethoxyflavone-2′-O-glucoside	16.69	491.1220	491.1195	C_23_H_24_O_12_	Flavone
9	Trihydroxyoctadecenoic acid	17.17	329.2379	329.2334	C_18_H_34_O_5_	Fatty acid derivative
10	Deoxygeniposidic acid	17.33	357.1155	357.1191	C_16_H_22_O_9_	Iridoid glucoside
11	Coniferyl ferulate	17.64	355.1225	355.1187	C_20_H_20_O_6_	Monolignol cinnamate
12	Methyltyrosine	19.48	194.0854	194.0823	C_10_H_13_NO_3_	Amino acid derivative
13	3-hydrossityrosol heptanoate	21.58	265.1513	265.1445	C_15_H_22_O_4_	Phenolic compound
14	Decyl gallate	25.41	309.1775	309.1708	C_17_H_26_O_5_	Phenolic compound
15	Hydroxyoctadecadienoic acid	25.89	295.2321	295.2279	C_18_H_32_O_3_	Fatty acid derivative
16	Hydroxylinolenic acid	26.20	293.2165	293.2122	C_18_H_30_O_3_	Fatty acid derivative
17	Dihydroxyoctadecadienoic acid	26.20	311.2273	311.2228	C_18_H_32_O_4_	Fatty acid derivative
18	Dihydroxyoctadecenoic acid	26.76	313.2421	313.2384	C_18_H_34_O_4_	Fatty acid derivative
19	Gingerol	27.06	293.1826	293.1758	C_17_H_26_O_4_	Phenolic compound
20	Dihydroxypropyl tetradecanoate	27.81	337.2091 ^b^	337.2151	C_17_H_34_O_4_	Fatty acid derivative
21	Olean-12-ene-3,7,15,21,23,28-hexol	27.89	551.3639 ^a^	551.3590	C_30_H_50_O_6_	Triterpenoid
22	Linoleic acid	28.34	279.2361	279.2330	C_18_H_32_O_2_	Fatty acid
23	Linoleyl 3-hydroxytyrosol	28.87	415.2906	415.2854	C_26_H_40_O_4_	Phenolic compound
24	Oleic acid	29.22	281.2521	281.2486	C_18_H_34_O_2_	Fatty acid
25	Palmitoyl 3-hydroxytyrosol	29.48	391.2895	391.2854	C_24_H_40_O_4_	Phenolic compound
26	Oleyl 3-hydroxytyrosol	29.67	417.3054	417.3010	C_26_H_42_O_4_	Phenolic compound
27	Oleanene-tetrol	30.18	519.3732 ^a^	519.3691	C_30_H_50_O_4_	Triterpenoid
28	Stearic acid	30.58	283.2676	283.2643	C_18_H_36_O_2_	Fatty acid
29	Palmitic acid	30.79	255.2369	255.2330	C_16_H_32_O_2_	Fatty acid
30	Octadecanoyl-glycerol	32.08	393.2829 ^b^	393.2777	C_21_H_42_O_4_	Fatty acid derivative
31	Palmitoyl 3-hydroxytyrosol isomer	32.25	391.2906	391.2854	C_24_H_40_O_4_	Phenolic compound

^a^ formiate; ^b^ chloride.

## Data Availability

Data presented in this study are available in this manuscript.

## Abbreviations

COX2	Cyclooxygenase 2
CIA	Collagen-induced arthritis
DMARDs	Disease-modifying anti-rheumatic drugs
DPPH	1,1-diphenyl-2-picrylhydrazyl
EVOO	Extra virgin olive oil
H_2_DCFDA	2′,7′-dichlorodihydrofluorescein diacetate
IL-1β	Interleukin-1β
IRD	Inflammatory rheumatic disease
IκB	Inhibitor of NF-κB
IKK	IκB kinase
LPS	Lipopolysaccharide
MnSOD	Manganese superoxide dismutase
MTS	3-(4,5-dimethylthiazol-2-yl)-5-(3-carboxymethoxyphenyl)-2-(4-sulfophenyl)-2H-tetrazolium
NF-κB	Nuclear factor kappa-light-chain enhancer of activated B cells
Nrf2	NF-E2-related factor 2
PBMCs	Peripheral blood mononuclear cells
PE-EVOOs	Polyphenol-enriched extracts
ROS	Reactive oxygen species
Th1	T helper 1
Th17	T helper17
TNF-α	Tumor necrosis factor-α
